# In Good Company? Perception of Movement Synchrony of a Non-Anthropomorphic Robot

**DOI:** 10.1371/journal.pone.0127747

**Published:** 2015-05-22

**Authors:** Hagen Lehmann, Joan Saez-Pons, Dag Sverre Syrdal, Kerstin Dautenhahn

**Affiliations:** School of Computer Science, University of Hertfordshire, College Lane, Hatfield, Hertfordshire, AL10 9AB, United Kingdom; Duke University, UNITED STATES

## Abstract

Recent technological developments like cheap sensors and the decreasing costs of computational power have brought the possibility of robotic home companions within reach. In order to be accepted it is vital for these robots to be able to participate meaningfully in social interactions with their users and to make them feel comfortable during these interactions. In this study we investigated how people respond to a situation where a companion robot is watching its user. Specifically, we tested the effect of robotic behaviours that are synchronised with the actions of a human. We evaluated the effects of these behaviours on the robot’s likeability and perceived intelligence using an online video survey. The robot used was Care-O-bot3, a non-anthropomorphic robot with a limited range of expressive motions. We found that even minimal, positively synchronised movements during an object-oriented task were interpreted by participants as engagement and created a positive disposition towards the robot. However, even negatively synchronised movements of the robot led to more positive perceptions of the robot, as compared to a robot that does not move at all. The results emphasise a) the powerful role that robot movements in general can have on participants’ perception of the robot, and b) that synchronisation of body movements can be a powerful means to enhance the positive attitude towards a non-anthropomorphic robot.

## Introduction

Research in social robotics and Human-Robot Interaction (HRI) has recently focused on building robotic companions capable of fulfilling a range of assistive functions [1] including healthcare and support for elderly people in their own homes. This development is also reflected in the increasing EU funding for projects specifically dedicated to companion robots [2, 3, 4]. One of the reasons for this process is the worldwide demographic change [5]. The percentage of elderly people in our societies has been increasing during the last decades and will continue to increase for the foreseeable future. This confronts our social and health systems with financial difficulties. One solution could be a combination of companion robots and smart home technology in private households to enable elderly people to live independently for longer in their own homes.

Companion robots could be used to remind people to drink fluids or to take their medicine regularly, keep them company and to alarm care personnel in case of potentially dangerous situations like injuries due to falling. In order for robots to be consented to in such scenarios, they need to be able to interact in a meaningful, predictable, and socially acceptable manner with their human users (see definition of companion robots in Dautenhahn, 2007 [1]).

At the University of Hertfordshire we have been studying since 2004, as part of the EU projects Cogniron [6], LIREC [7] and ACCOMPANY [2], how robot home companions should behave towards and in the presence of people [8, 9, 10, 11, 12]. One particular question that has emerged during this research concerns how robots should behave during time periods in which the user is engaged in activities like reading, watching TV, and preparing a meal. Without a concrete task to do, the robot could return to its charging station, however, in its role as a *companion* robot, shall it ‘keep company’ with its user for example, in a pet like way, i.e. by standing nearby and non-intrusively observing and non-verbally responding to the user’s behaviours, ready to engage if the user has finished her current activity? How would people perceive such behaviours from a companion robot? Would ‘being watched’ by a machine be perceived as threatening?

In order to address these issues, we designed a study whereby a non-anthropomorphic robot, the Care-O-bot3, watched a person being engaged in a physical manipulation task. The behaviour of the robot towards the person was designed based on the concepts of head gaze, which plays an important role during human-human interaction and joint attention. We tested how different forms of synchronised head gaze movements were perceived, hypothesizing that the ability for a robot to appear engaged in tasks that normally require joint attention in human-human interaction would facilitate the robot’s social acceptance and perception as a social entity.

The article is structured as follows. First, we discuss the relevant background information and motivate our research questions. Next, we describe our experimental setup, including the robotic platform we used, and provide an overview of our methods. We then present our results and discuss them in relation to other findings in the research field, acknowledging limitations of the work and pointing out directions for future research.

## Background

### Anthropomorphisation

Many of the currently available social robots are to a certain degree anthropomorphic [13, 14, 15, 16]. If they are not humanoid they usually have at least a part that loosely resembles a ‘head’ with facial features like ‘eyes’. The reason for this is that for robots incorporating such features it is easier to facilitate social interactions with humans, because their appearance helps them to directly emulate aspects of human-human social interaction dynamics [17]. Nevertheless, humanlike appearance by itself does not ensure comfortable HRI. Another very important aspect is the movement of the robot. The more naturalistic the movements of the robot are, the more comfortable will the interaction be [18]. Unnatural movements and behaviours, specifically in anthropomorphic robots, cause them to fail the expectations triggered by their appearance and to fall into the (hypothesised) Uncanny Valley [19, 20, 21].

One way to avoid this effect would be to equip robots with sensors and actuators that allow them to flexibly exhibit appropriate human-like behaviour. Unfortunately this would require deep models of cognition and emotion, which at the current state of technology cannot easily be implemented yet. A practical alternative is to provide the robots with external expressions typical of humans during a social interaction. These expressions are typically composed of body movements, gestures and speech. Our decision to use body movements, specifically movements resembling *head gaze*, was owed to the physical affordances of the Care-O-bot3 and the role gaze following behaviour plays for humans during cooperative, mutualistic social interactions [22].

Gaze is the main non-verbal source of social information between individuals, who understand each other as intentional agents with emotional states. In order to develop artificial systems with which humans feel comfortable interacting, it is necessary to consider the mechanisms of human gaze behaviour and its related movements [23].

### Synchronisation of behaviour

The perception of appropriateness of gaze during social interaction depends strongly on timing. The synchronisation of ones own movements with the actions and movements of others is as important as their naturalistic appearance for meaningful and comfortable interaction [24, 25, 26]. There are different ways behavioural synchronisation can be used in experiments—movement synchronisation, synchronisation of direction (opposite—same), and synchronisation focused on a specific object or location. It could be argued that positive, object centred synchronisation represents a form of object centred joint attention. In developmental psychology this form of joint attention is seen as a prerequisite for social learning [27, 28] during ontogenetic development. It represents a very basic mechanism that allows humans to interact goal directed and purposefully.

When employing a robotic system with a set of limited torso movements in situations involving social interaction, it is important to consider the synchronization of these non-verbal behaviours with the behaviours of the user. In HRI synchronized movements can be also referred to as congruent or contingent movements [29]. The synchronization can be positive or negative. ‘Positive’ means in this context that the robot follows the movements and actions of the human, generating the impression of being attentive, and ‘negative’ means the robot is doing the exact opposite movements of the human.

### Research Questions and Expectations

Our study aims to answer three research questions:

- Research Question 1 (RQ1): Are synchronised movements of a non-anthropomorphic robot sufficient to influence participants’ perception of the robot?
- Research Question 2 (RQ2): If RQ1 can be positively answered, what role does the direction of the synchronisation play?
- Research Question 3 (RQ3): Are the possible effects found in RQ1 and RQ2 influenced by factors like age, gender and prior experience with robots?

Concerning RQ1, we expected that humanlike movements such as synchronised head gaze following would induce an emotional reaction in the user, even when confronted with a robot without a clearly distinguishable head. This expectation is based on the hypothesis that humanlike movements are more important than humanlike appearance [19].

In case of RQ2, we expected that the robot would be perceived more friendly and likeable, when exhibiting movements that are positively synchronised with the actions of the user. We hypothesise that even with its limited social interaction features, Care-O-bot3 will be perceived as likeable and friendly if it exhibits certain behaviours (i.e. “head” gaze) in positive synchronisation with the actions of the user.

For RQ3 we expected the potential effects to be fairly robust with regard to demographic factors. Prior research with other robotic platforms and different settings has shown only little influence [30, 31, 32].

## Methods

### Ethics statement

The research was approved by the University of Hertfordshire’s ethics committee for studies involving human participants (under protocol no. a1112/161). The participants provided their informed consent before seeing the videos and responding to the questions.

### Materials

We conducted an online survey, in which we presented three pre-recorded one-minute videos, in a random order, each followed by a questionnaire. The survey consisted of three parts (see Annex 1 for a full version). The first part briefly introduced the tasks and included the ethics approval and consent form. The second part contained demographic questions and the last part included the actual study showing three video conditions in a randomised order followed by the evaluation tools. We have used the Video-based HRI (VHRI) methodology reliably in several human-robot interaction studies in the past [9, 33, 34, 35]. In a direct comparison of live HRI and Video-based HRI we found comparable results [36, 37].

**Fig 1:**
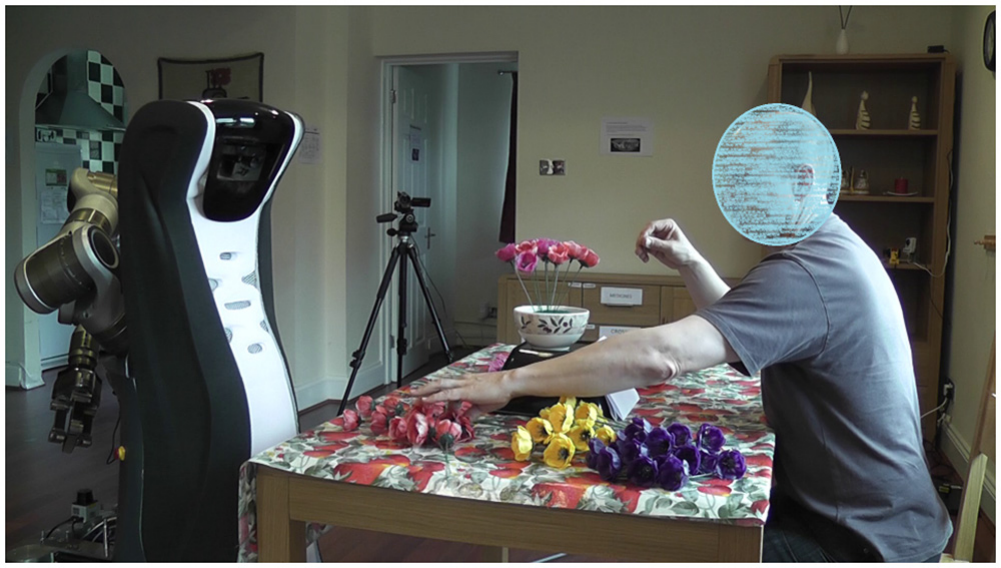
Experimental setup from side perspective showing the robot and the ‘user’.

**Fig 2:**
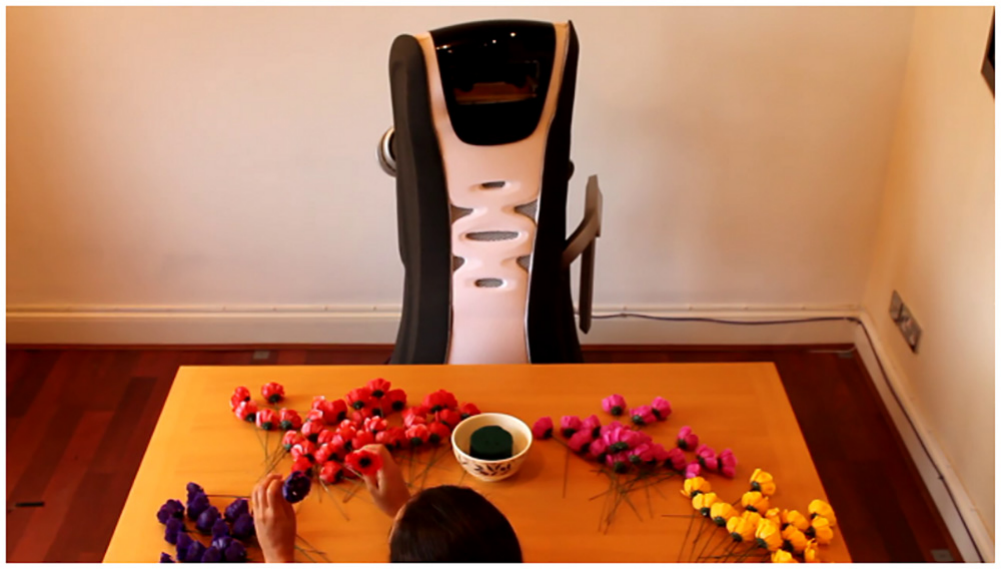
Final experimental setup showing the “over-the-shoulder” perspective.

Small-scale pilot and validation studies were carried out in order to determine the most appropriate camera perspective for the final online survey [38]. We tested two different settings. For the first setting we chose a camera angle that allowed observing the entire interaction and to see both the robot and the ‘user’, i.e. the actor shown in the video. For the second setup an “over-the-shoulder” perspective was chosen, allowing the observation of the movements of the robot from the front and only the movements of the hands of the user (see Figs 1 and 2). The results from the pilot and validation studies showed that participants were capable to distinguish the different robot behaviours and rate them accordingly in both perspectives. For the final study, we decided to use the over-the-shoulder perspective considering that it would allow for a much better control of confounding variables, i.e. participants were able to be more focused on the movements of the robot, without being distracted by contextual information such as the gender, age or ethnic background of the person shown in the video. The final perspective can be seen in Fig 2.

**Fig 3:**
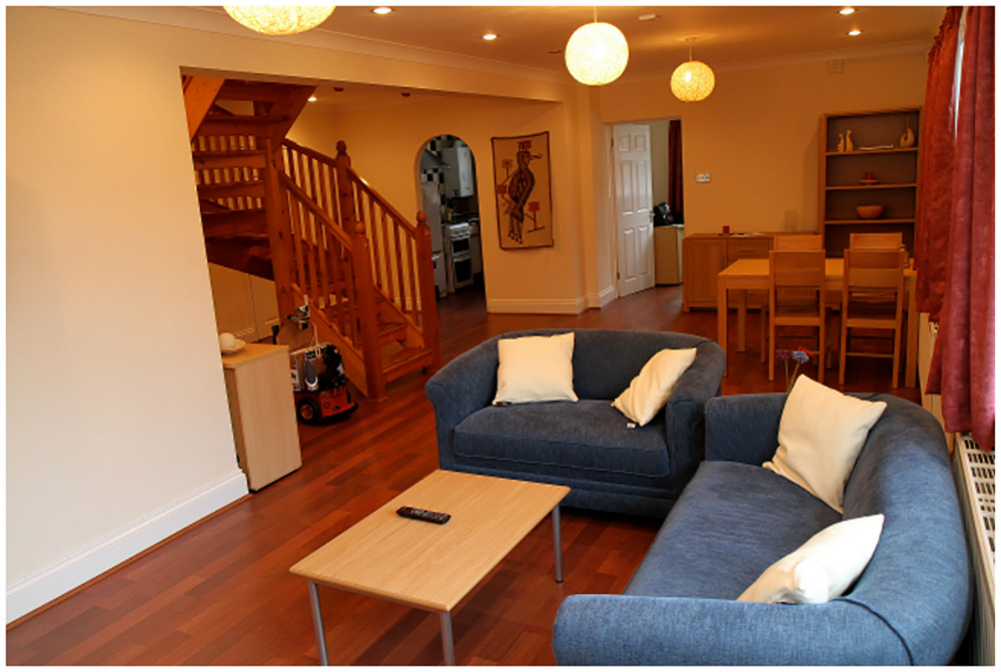
“Robot House” at the University of Hertfordshire.

The videos were produced in the living room of the University of Hertfordshire’s Robot House, a facility dedicated to HRI research in a realistic, domestic environment. The Robot House has the appearance of an ordinary, fully-furnished, British suburban house (Fig 3). The house is populated with different robot companions, which are integrated into a sensor network in order to create a smart home environment.

The robot involved in this research was Care-O-bot3, a non-anthropomorphic service robot developed by the Fraunhofer Institute for Manufacturing, Engineering and Automation [39]. This robot is an omnidirectional platform equipped with a 7 degrees of freedom (DOF) KUKA lightweight arm and the 7 DOF Schunk Dexterous Hand with integrated tactile sensors. Its torso is mounted on a moveable platform, the KUKA industrial arm is located on its back and it has an extendable tray on the front. The robot also has two cameras on the upper end of the torso, a set of differently coloured LED lights on its front and synthetic text to speech. Due to its non-anthropomorphic appearance it cannot generate complex human-like gestures—it lacks a face, a clearly distinguishable head, a humanoid body and arms and hands for gesturing. Nevertheless, most of Care-O-bot3’s mobility is based on the torso which allows it to perform basic movements such as bending forward and twisting the torso to the sides. We decided to use these turn and forward motions during the experiment, to simulate gaze following behaviour.

### Measures

Our analysis of the data is based on two standardised, validated measures. The first is the Godspeed Questionnaire created and validated by Bartneck et al. [40]. The second is the Inclusion of Other in Self (IOS) scale, as described in Aron et al. [41].

#### The Godspeed Questionnaire

The Godspeed questionnaire was devised as a HRI-specific measure of participant perceptions across several dimensions, where each dimension is addressed using a set of semantic differential scale [40]. Due to the constraints of an online study in which we needed to have a brief questionnaire, we chose 2 dimensions from this questionnaire that are most relevant to the present study, Likeability and Perceived Intelligence. The set of semantic pairs for each dimension is included in Table 1. Each dimension was treated as an interval scale in accordance with the assumptions of classical test theory [42].

**Table 1:** Semantic pairs for Godspeed Scale dimensions used.

*Likeability*	*Perceived Intelligence*
Dislike—Like	Incompetent -Competent
Unfriendly—Friendly	Ignorant—Knowledgeable
Unkind—Kind	Irresponsible—Responsible
Unpleasant—Pleasant	Unintelligent—Intelligent
Awful—Nice	Foolish—Sensible

#### Inclusion of Other in Self Scale

**Fig 4:**
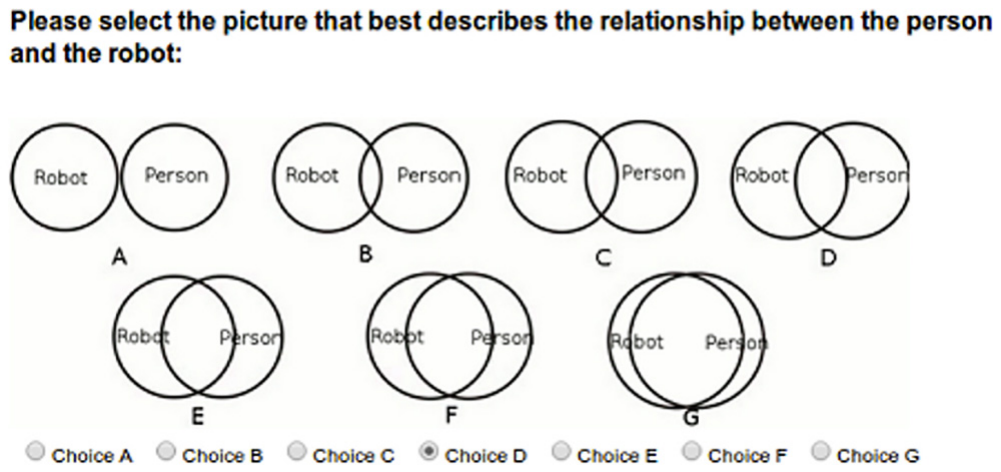
IOS Scale.

The IOS Scale in this study was used, based on Aron et al. [41], as a pictorial scale of closeness in which participants can describe their relationship with an ‘other’ by selecting a picture from a set of Venn-like diagrams which depict two circles that overlap to differing degrees. The overlapping area of the different circles changes linearly from each picture to the next, and can be compared visually by the participant, in terms of absolute degrees of overlap rather than merely relative to the adjacent images. Because of this we treated participant responses to this scale as a seven-point interval scale [43]. The scale is presented below in Fig 4.

In addition to the experimental items in the questionnaires we collected demographic data from the participants including age, gender, prior experience with the robot Care-O-bot3 and with robots in general from the participants.

### Recruitment of participants

The call for participation in the survey was distributed via different mailing lists, i.e. the robotics-worldwide mailing list, the euron-dist mailing list and the PHILOS-L mailing list. During the 12 days the survey was open 301 participants started the questionnaire. Out of those 119 completed all questions. The remaining 182 were excluded as a result of missing or repetitive answers.

### Recording of the videos

The videos used in the online survey showed an actor arranging coloured plastic flowers to a bouquet and being observed passively by the robot. In the online survey we used three different experimental conditions in order to test our research questions. The robot’s engagement behaviour differed in the three experimental conditions.

**Fig 5:**
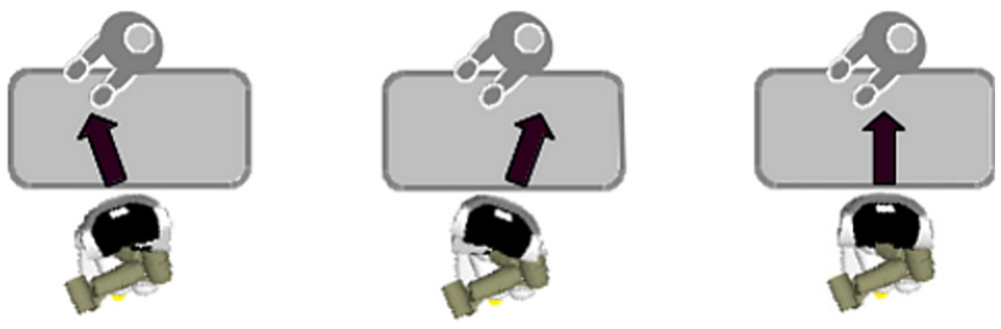
Experimental Setup—Showing the movements of the actor and the respective movements of the robot for each condition. (Left to right: positive synchronised behaviour, negative synchronised behaviour, no movement).

At the start of the video the flowers were laid out in front of the actor on a table. A vase with floral foam was located on the table. The actor was asked to arrange the flowers freely in the vase. The effectiveness and suitability of this task was tested prior to the study with two resident artists at University of Hertfordshire. During a weeklong co-habitation experiment the artists participated in the development of the task and in an initial set of test runs [44]. Fig 5 shows the final layout of the experiment.

### Experimental conditions

1. Condition 1 (Positive Synchrony). In the first experimental condition the robot exhibited positive synchronization by following the actor’s actions towards the objects on the table accordingly. Wherever the object of interest for the actor was (flower on the table, vase in the center in front of the user) the robot followed the movements of the human with its “gaze”, giving the appearance to be engaged and interested in what the actor was doing. With this condition we wanted to examine the effect of this behaviour on the perception of the robot’s perceived sociability.
2. Condition 2 (Negative Synchrony). In the second experimental condition the robot exhibited negative synchrony (In the initial phase of the experiment we considered using a random movement pattern in condition 2. Besides procedural difficulties—we noticed in the pre-tests that it was very distracting for the actor and hence very difficult to keep the random movement truly random-, it would have also not allowed us to test our second research question.). It moved its “gaze” always opposite to where the user was moving. If the user was positioning the flower in the vase in the middle of the table, the robot looked left or right, giving the appearance of avoiding paying attention to what the user was doing. With this condition we wanted to test whether the fact that the robot was moving, even though it appeared not to be engaged with the user, would have a positive effect compared to the control condition. Previous research has shown that animated artificial agents are in general anthropomorphized and ascribed social roles and behaviours [45, 46, 47].
3. Condition 3 (Control). In the control condition the robot was not moving at all and was “looking” straight forward during the entire experiment. This condition controlled for the overall effect of robot movement vs. non-movement.

The participants were not given any information about the purpose of the study or the reasons behind the robot’s behaviour.

## Results

### Characteristics of the Sample

The sample consisted of 119 participants. The mean age was 35.28, ranging from 22 to 76, and with a median age of 32. There were 36 females in the sample, and 83 males.

### Results for Godspeed Questionnaire Subscales

In order to address research question 1 and 2, the differences in participant ratings of the robot along both the two subscales of the Godspeed Questionnaire as well as their responses to the Inclusion of Other in Self scale between the different conditions were examined.

#### Reliability measures

To ensure that the sets of pairs described in Table 1 could be used as scales, the internal consistency of the two subscales was assessed using Cronbach’s alpha. The high Cronbach’s α across the three conditions shown in Table 2 suggested that we could proceed treating these two dimensions as interval scales as proposed by Bartneck et al. [40].

**Table 2:** Godspeed questionnaire reliability.

Condition	Dimension	alpha
1	Likeability	0.87
	Perceived Intelligence	0.86
2	Likeability	0.93
	Perceived Intelligence	0.88
3	Likeability	0.91
	Perceived Intelligence	0.87

The subscale scores for each dimension for each condition was calculated and all subsequent analyses of the Godspeed subscales were done on the dimension subscales rather than the individual items.

The descriptive statistics for Likeability are presented in Table 3.

**Table 3:** Descriptive statistics Likeability.

Condition	Variable	Mean (SD)	Median	95%CI	t(p)
Condition 1	Likeability	3.47 (0.6)	3.6	3.36–3.58	8.48 (<.01)
Condition 2	Likeability	2.56 (0.81)	2.6	2.41–2.71	-5.93 (<.01)
Condition 3	Likeability	2.2 (0.71)	2.2	2.07–2.33	-12.24 (<.01)

The results suggest that for the Likeability dimension participants overall scored the robot higher than a “neutral” score of 3 only in condition 1, while the participants scored the robot lower than this “neutral” score in both condition 2 and 3. The effect of condition on participant ratings along this dimension was assessed using a repeated measures ANOVA. The repeated measures ANOVA found a significant effect for Condition (F (2, 236) = 147.12, p = 0, partial *η* ^2^ = 0.55.

Post-hoc tests presented in Table 4, suggest that there were significant differences between all three conditions, with Condition 1 receiving the highest scores, followed by Condition 2 and Condition 3 receiving the lowest scores.

**Table 4:** Post-hoc tests for Likeability.

Pair	Mean Difference	95% CI of Diff.	t(df)	p
Con1Liking—Con2Liking	0.91	0.75–1.07	11.38 (118)	<.01
Con1Liking—Con3Liking	1.27	1.13–1.41	18.17 (118)	<.01
Con2Liking—Con3Liking	0.36	0.21–0.52	4.59 (118)	<.01

The results presented in Table 5 suggest that for the Perceived Intelligence dimension participants would only score the robot higher than a “neutral” score of 3 only in Condition 1, and lower than this “neutral” score in Condition 2 and 3. The effect of condition on participant ratings along this dimension was likewise assessed using a repeated measures ANOVA. The repeated measures ANOVA found a significant effect for Condition (F (2, 236) = 114.53, p = 0, partial *η* ^2^ = 0.49.

**Table 5:** Descriptive statistics for Perceived Intelligence.

Condition	Variable	Mean(SD)	Median	95%CI	t(p)
Condition1	Perceived Intelligence	3.13 (0.67)	3.2	3.01–3.25	2.15 (0.03)
Condition2	Perceived Intelligence	2.42 (0.74)	2.4	2.29–2.56	-8.51 (<.01)
Condition3	Perceived Intelligence	2 (0.69)	2	1.87–2.12	-15.92 (<.01)

Post-hoc tests shown in Table 6 found significant differences between all three conditions, suggesting that participants rated the robot highest along this subscale in Condition 1 followed by Condition 2 and finally by Condition 3.

**Table 6:** Post-hoc tests for Perceived Intelligence.

Pair	Mean Difference	95% CI of Diff.	t(df)	p
Con1Intell—Con2Intell	0.71	0.55–0.86	8.91 (118)	<.01
Con1Intell—Con3Intell	1.13	0.98–1.29	14.34 (118)	<.01
Con2Intell—Con3Intell	0.43	0.29–0.56	6.27 (118)	<.01

### Results for Inclusion of Other in Self Scale

While the results in Table 7, suggest clear differences between the conditions, all the mean median scores are below the “middle” rating of 4. The repeated measures ANOVA found a significant effect for Condition (F (2, 236) = 130.29, p = 0, partial *η* ^2^ = 0.52.

**Table 7:** Descriptive statistics for the IOS.

Condition	Variable	Mean (SD)	Median	95%CI
Condition 1	IOS	2.91 (1.25)	3	2.68–3.13
Condition 2	IOS	1.92 (1.06)	2	1.72–2.11
Condition 3	IOS	1.17 (0.53)	1	1.07–1.26

The post-hoc tests shown in Table 8 for the IOS scale also suggest the same relationship between conditions as that found for the other two measures, i.e. with highest scores for Condition 1 followed by Condition 2 and finally by Condition 3.

**Table 8:** Post-hoc tests for the IOS.

Pair	Mean Difference	95% CI of Diff.	t(df)	p
IOS1Number—IOS2Number	0.99	0.74–1.24	7.9 (118)	<.01
IOS1Number—IOS3Number	1.74	1.53–1.95	16.45 (118)	<.01
IOS2Number—IOS3Number	0.75	0.55–0.94	7.59 (118)	<.01

### Impact of Demographics

In order to address Research Question 3, the relationships between demographic factors and prior experience of robots, and responses along the Godspeed subscales and the IOS scale were examined using a series of Spearman's correlations. This analysis found no significant relationships.

## Discussion

### Findings

The results largely confirm our initial hypotheses. We expected the robot to be rated most likeable in the condition in which its behaviour was synchronised with the behaviour of the actor. In the condition in which Care-O-bot3 exhibited positive synchronisation it was rated most likeable and intelligent, followed by the condition in which the robot exhibited negative synchronisation. We could further show that people rated an animated robot more likeable than the same robot not moving. This concurs with previous HRI findings [48] and with studies in developmental psychology research, which show that humans start at a very early age to ascribe goals and mental states to moving agents [49, 50]. In Condition 3, in which the robot did not move at all, it was rated the least likeable and least intelligent. The findings of the Inclusion of Others in Self Scale follow the same pattern. The participants rated the relationship between the actor and the robot closest in the condition in which the robot’s behaviour was synchronised with the actions of the human.

We found no correlations between age and perceived intelligence, likeability or the rating of the IOS scale. We also found no correlation between prior experience with robots in general or exposure specifically to the Care-O-bot3 with any of the measurements. In contract to the findings of other studies [51, 52] we found no correlations between the gender of the participants and our measurements.

The effect that the robot is perceived more positive, even when it is exhibiting behaviour that could be interpreted as ‘avoidance’, is a key finding of our study, providing on the one hand interesting insights in the human perception of social robots and agents, and on the other hand has practical consequences for designers of robot home companions. It seems that exhibiting behaviour that can be interpreted as coherent and goal-directed, even if it does not conform to what would be expected of socially engaging behaviour, facilitates the human propensity to ascribe intentions to agents (objects).

One of the first examples demonstrating this effect was introduced by Heider and Simmel [45]. They presented a short cartoon involving two triangles and a dot moving inside and outside a square to participants. Their results indicated that depending on the movement of the geometric shapes, people started to ascribe not only intentions but also personality characteristics to them. Tremoulet and Feldman [53] demonstrated that a single moving object can be perceived as being ‘alive’, depending on the variations in speed and directions of the motion. Pantelis et al. [54] have shown that the attribution of intentionality also applies to autonomous agents interacting with a virtual environment. Gergely et al. [55] demonstrated that 12-month-old pre-verbal children already interpret agent (objects) “acting” seemingly goal directed as having intentions, and according to Meltzoff [56] 18-month-old children are already capable of understanding the intention of others correctly. More recently, Ma and Xu [57] have shown that pre-verbal infants by about 9–10 months of age infer the presence of an intentional agent from the perception of regularity in a visual display. These results from developmental psychology research indicate how deeply rooted the propensity of understanding the goal directed movements of agents as being intentional is in human ontogeny.

In the last 20 years this has been of increasing interest in the HRI community. The research on the social aspects of HRI focuses on the expressivity that proxemics, movements, gestures, and postures can give to social robots. It is concerned with how to integrate robotic agents into human social ecologies, including robotic agents built to accomplish tasks in education, entertainment, assistance, mediation in therapeutic relations, and rehabilitation contexts. The focus is on how to enhance the capabilities of robots to interact efficiently in the social domain they are built for, in order to improve their social acceptance [58].

Dautenhahn [59] identified 6 factors that facilitate the human perception of robots as intentional agents. Amongst these factors are goal-directed behaviour, and synchronisation and interactivity. Our findings show that even without interactivity people prefer a robot exhibiting seemingly synchronised behaviour. This is arguable a result of interpreting the robot’s movements as active avoidance and therefore being intentional. Using the human propensity for ascribing agency and intentionality to animated agents (objects), even in situations in which the robot is not actively participating in a given task, might be crucial for the successful introduction of robot companions into private homes and possibly other human-inhabited environments.

The results also have possible practical considerations for designing socially acceptable behaviours for robot home companions. A moving robot, even if it is moving in a way that is not following the expected ‘social behaviour’, and this might include a robot that is moving but malfunctioning, or a robot where the behaviour policies, perceptual or movement abilities are limited, may still be perceived more positively than a robot that is not moving at all. Thus, making a robot move seems to be a crucial requirement for a home companion robot. These results from our study are supported by previous research on robots with ‘idle movements’, such as blinking or gaze avoidance, which have been shown to be perceived more positively compared to robots without such movements. The specific use of robot movements such as gaze, nodding, blinking, and human-robot movement contingency has been discussed in the context of specific communicative functions in human-robot interaction (see e.g. [60–64]). However, our results indicate that the mere introduction of such movements to a robot, even when not yet functioning at the level of complexity we find in humans, may already increase participant’s acceptance and positive perception of the robot, compared to a robot lacking such movements at all. Future research needs to investigate the interrelationships between robot and human movements, non-verbal cues and the attribution of agency in more detail.

### Limitations of study

During the experiment the robot was a passive observer. There was no direct communication or interaction between the user and the robot, however in the positive synchronisation condition the robots behaviour suggested joint attention towards the objects the human user was manipulating during the course of the experiments. The robot was also based in close proximity to the human user, which arguably created the impression of a social interaction between the robot and the user from the perceptive of an outside observer. In this specific sense we speak of an interaction scenario.

Our call for participation received 301 replies within 12 days. Being able to reach this many participants in such a short time is an argument in favour for using an online survey. There are arguments for both using video material to rate behaviour and for exposing the participants to the actual robot in a real world scenario. It has been shown in the past that the actual physical presence of the participant during the experimental scenario with a robot can have a strong effect on the perception and rating of behaviours exhibited by the involved robot [65, 66]. On the other hand there are many video studies with virtual artificial agents on screens that illustrate that watching behaviour in a video is a valid method to create reliable results [67, 68].

Another potential source of bias in our results is the selection of mailing lists for the call for participation. We selected two robotic mailing lists and a mailing list mainly read by philosophers and psychologists. This might have had an effect on the results. Even though the data suggests a reasonably well contribution of prior experience with robots, the sample was very likely not representative for the general population. Access to these mailing lists is usually restricted to academics and students. Furthermore a positive reply to our request suggests an interest and possibly a positive predisposition towards the topic. However, this is a problem social sciences and HRI research involving human volunteers as participants is facing in general. The use of mailing list makes this problem more pronounced due to the pre-selection of the users of these mailing lists. For further HRI research using online video studies other forms of distributing the call for participation, e.g. social media like Facebook, could be involved.

### Summary of Hypotheses and Implications

The main research intent of this study was to investigate how participants judge a scenario where a robot is watching the activities of a person. Specifically, we were interested in the effects of different forms of behavioural synchronisation exhibited by a non-anthropomorphic robot on its “Likeability” and “Perceived Intelligence”, and to evaluate its IOS Scale rating by an outside observer. We showed that positive behaviour synchronisation resulted in the highest ratings, followed by negative synchronisation, which was followed by no movement at all. Our assumptions from RQ1 and RQ2 were confirmed. With respect to RQ3 we found no correlations concerning demographics and background of the participants.

### Future work

As the discussion illustrates there are different directions that future research based on this study could take. The results of our study backup the iterative use of online video surveys in HRI to support rapid prototyping of robotic behaviours and HRI scenarios. Further research will need to examine the specificities of potential attention (idle) behaviours for robotic companions. A variety of different scenarios can be tested in order to generate a database for these types of situations. Live HRI studies, with participants observing a robot watching a person, and with participants themselves being watched, need to validate the results from the video studies.

## Conclusion

The aim of our study was to test the effects of different behavioural interaction patterns of Care-O-bot3 on its perceived likeability, perceived intelligence and involvement, in a situation where a robot watches a person. We tested specifically how people would react to different levels of behavioural synchronisation between the actions of a human and the robot. Our results showed that the positive perception of the robot could be enhanced if the robot follows the actions of its user. We showed that people perceive a robot that moves in a non-synchronised way as more likeable, compared to an inactive robot.

The results show that, for a companion robot in people’s own homes, even in situations where the robot is not directly involved in the tasks, its behaviour has a significant impact on the overall user experience of the robot.

## Supporting Information

S1 Supporting Information Compressed/ZIP File Archive. (ZIP)
