# Supplementary material for: In Good Company? Perception of Movement Synchrony of a Non-Anthropomorphic Robot
Source: PLoS One. 2015 May 22;10(5):e0127747. doi: 10.1371/journal.pone.0127747 (PMC4441426; doi:10.1371/journal.pone.0127747)
Supplement: S1 Supporting Information — (ZIP) [file pone.0127747.s001.zip › Supporting Information - CompressedZIP File Archive/StatisticalOverview.pdf]

Statistics Overview

Introduction

This documents outlines the statistical analysis of the paper. It has the appropriate R-code available for replicability of the analysis. It is distributed wth a copy of the file “ContingentData.csv”, which contains the raw data. It is also distributed with a .rmd file used by RStudio, but which is also readable in any text editor. We hope that it will address some of the points raised by reviewer 1 regarding our choices for the statistical analysis of the data.

Housekeeping

Libraries

This includes the libraries needed for the analysis.

```
library(knitr)
```

```
## Warning: package 'knitr' was built under R version 3.0.3
```

```
library(coefficientalpha)
library(Rmisc)
```

```
## Warning: package 'Rmisc' was built under R version 3.0.3
## Warning: package 'plyr' was built under R version 3.0.3
```

```
library(car)
```

```
## Warning: package 'car' was built under R version 3.0.3
```

```
library(reshape2)
```

```
## Warning: package 'reshape2' was built under R version 3.0.3
```

```
library(ggplot2)
```

```
## Warning: package 'ggplot2' was built under R version 3.0.3
```

Functions

The following are ad-hoc functions created for the analysis, they are not commented, but they should not be complex enough for this to be a problem.

```
Reliability <-function(variables, data, name){
  PertData <- data[variables]
  Crondat <- cronbach(PertData)
  PertMatrix <- matrix(ncol=3, nrow=1)
  PertMatrix[1,]<-  c("", name ,round(Crondat$alpha,2))
```

```
  return(PertMatrix)
}


SequenceMatrix <- function(variables, data, Names){
  seqmatrix <- matrix(nrow=1, ncol=6)
  colnames(seqmatrix)<- c("Condition","Variable","Mean (SD)", "Median", "95%CI", "t(p)")
  seqmatrix[1,]<-c("", "", "", "", "", "")
  for (i in 1:length(variables)){
    PertMatrix <- matrix(ncol=6, )

    pertvar <- variables[i]
    pertdat <- data[pertvar]
    pertdat <- as.numeric(unlist(pertdat))
    PertMatrix[1,1]<-paste("Condition", i)
    PertMatrix[1,2]<-Names[i]
    PertMatrix[1,3]<-paste(round(mean(pertdat),2), "(",round(sd(pertdat),2),")")
    PertMatrix[1,4]<-median(pertdat)
    PertMatrix[1,5]<-paste(round(CI(pertdat)[3],2),"--",round(CI(pertdat)[1],2))
    PertMatrix[1,6] <-paste(round(t.test(pertdat, mu=3)$statistic,2),
                        "(", round(t.test(pertdat, mu=3)$p.value,2),")")
    seqmatrix<-(rbind(seqmatrix, PertMatrix))


  }
  return(seqmatrix)
  }



Posthoc <-function(InterSpes, spesdat){

  PostMatrix <- matrix(ncol=5)
  PostNames <- c("Pair", "Mean Difference", "95% CI of Diff.", "t(df)", "p")
  colnames(PostMatrix) <- PostNames
  for (i in (1:(length(InterSpes)-1))){

    PerMatrix <- matrix(ncol=5, nrow=(length(InterSpes)-1))
    for (xi in (1:(length(InterSpes)-1))){
      HoldVar <- InterSpes[1]
      GoVar <- InterSpes[(xi+1)]
      A <- spesdat[HoldVar][,1]
      B <- spesdat[GoVar][,1]

      PerTest <- t.test(A,B, paired=T)
      PerMatrix[xi,1] <- paste(HoldVar, " - ", GoVar)
      PerDiff <- as.numeric(unlist(spesdat[HoldVar] - spesdat[GoVar]))
      PerMatrix[xi,2] <- round(mean(as.numeric(unlist(PerDiff))),2)
      PerMatrix[xi,4] <- paste(round(PerTest$statistic,2),
                         "(",round(PerTest$parameter,2),")", sep="")
      PerMatrix[xi,3]<- paste(round(CI(PerDiff)[3],2),"--",
                        round(CI(PerDiff)[1],2))
```

```
      PerMatrix[xi,5] <- paste(round(PerTest$p.value, 2))
    }
    PostMatrix <- rbind(PostMatrix, PerMatrix)
    InterSpes <- InterSpes[-1]

  }
  PostMatrix <- PostMatrix[-1,]
  return(PostMatrix)
}

IOSSequenceMatrix <- function(variables, data, Names){
  seqmatrix <- matrix(nrow=1, ncol=5)
  colnames(seqmatrix)<- c("Condition","Variable","Mean (SD)", "Median", "95%CI")
  seqmatrix[1,]<-c("", "", "", "", "")
  for (i in 1:length(variables)){
    PertMatrix <- matrix(ncol=5, )

    pertvar <- variables[i]
    pertdat <- data[pertvar]
    pertdat <- as.numeric(unlist(pertdat))
    PertMatrix[1,1]<-paste("Condition", i)
    PertMatrix[1,2]<-Names[i]
    PertMatrix[1,3]<-paste(round(mean(pertdat),2), "(",round(sd(pertdat),2),")")
    PertMatrix[1,4]<-median(pertdat)
    PertMatrix[1,5]<-paste(round(CI(pertdat)[3],2),"--",round(CI(pertdat)[1],2))

    seqmatrix<-(rbind(seqmatrix, PertMatrix))


  }
  return(seqmatrix)
  }

WilkPosthoc <-function(InterSpes, spesdat){

  PostMatrix <- matrix(ncol=5)
  PostNames <- c("Pair", "Mean Difference", "Z", "p", "r")
  colnames(PostMatrix) <- PostNames
  for (i in (1:(length(InterSpes)-1))){

    PerMatrix <- matrix(ncol=5, nrow=(length(InterSpes)-1))
    for (xi in (1:(length(InterSpes)-1))){
      HoldVar <- InterSpes[1]
      GoVar <- InterSpes[(xi+1)]
      PertTest <- wilcox.test(as.numeric(unlist(spesdat[HoldVar])),
                              as.numeric(unlist(spesdat[GoVar])),paired = T)
      Z <- qnorm(PertTest$p.value/2)
      PerMatrix[xi,1] <- paste(HoldVar, " - ", GoVar)
      PerDiff <- as.numeric(unlist(spesdat[HoldVar] - spesdat[GoVar]))
      PerMatrix[xi,2] <- round(mean(as.numeric(unlist(PerDiff))),2)
      PerMatrix[xi,3] <- round(Z,2)
      PerMatrix[xi,4] <- round(PertTest$p.value,2)
      B <- spesdat[GoVar][,1]
```

```
      PerMatrix[xi,5] <- round(Z/sqrt(length(B)),2) }
    PostMatrix <- rbind(PostMatrix, PerMatrix)

    InterSpes <- InterSpes[-1]

  }
  PostMatrix <- PostMatrix[-1,]
  return(PostMatrix)
}
```

Data

```
fulldata  <- read.table("ContingentData.csv", header=TRUE, sep=",")

#getting rid of the missing var
cleanvar <- names(fulldata) %in% c("X","X.1")
cleandata <- fulldata[!cleanvar]
cleandata <- subset(cleandata, cleandata$Age > 17) #(removing data from participants under the age of 1
```

Measures

This analysis is based on two standardised, validated measures. The first is the *Godspeed Questionnaire* created and validated by Bartneck et al. [1]. The second is the *Inclusion of Other in Self* (IOS) scale, as described in Aron et al.[2].

The Godspeed Questionnaire

The Godspeed questionnaire was devised as a Human-Robot Interaction specific measure of Participant perceptions across several dimensions, where each dimension is addressed using a set of *semantic differential* scales Due to the constraints of an online study in which we needed to have a brief questionnaire, we chose to only investigate 2 dimensions from this questionnaire, *Likeability* and *Perceived Intelligence*. The set of semantic pairs for each dimension is included in Table 1.

Likeability	*Perceived Intelligence*
Dislike - Like	Incompetent -Competent
Unfriendly - Friendly	Ignorant - Knowledgable
Unkind - Kind	Irresponsible - Responsible
Unpleasant - Pleasant	Unintelligent - Intelligent
Awful - Nice	Foolish - Sensible

Table 1: Semantic pairs for Godspeed Scale dimensions used.

[1] Bartneck, C., Kulic, D., Croft, E., & Zoghbi, S. (2009). Measurement instruments for the anthropomorphism, animacy, likeability, perceived intelligence, and perceived safety of robots. International journal of social robotics, 1(1), 71-81.

[2] Aron, A., Aron, E. N., & Smollan, D. (1992). Inclusion of Other in the Self Scale and the structure of interpersonal closeness. Journal of personality and social psychology, 63(4), 596.

Inclusion of Other in Self Scale

The IOS Scale used in this study was based on that described by Aron et al. as a pictorial scale of closeness in which participants can describe their relationship with an 'other' by selecting a picture from a set Venn-like diagarams which depicts two circles that overlap to differing degrees. The overlapping area of the different circles changes linearly from each picture to the next, and can be compared visually by the participant, in terms of absolute degrees of overlap rather than merely relative to the adjacent images. This lends support to the notion of treating participant responses to this scale a seven-point interval scale.[3] However the low number of points on this scale suggests that some participants may still treat as an ordinal scale.

The scale is presented below in Figure 1.

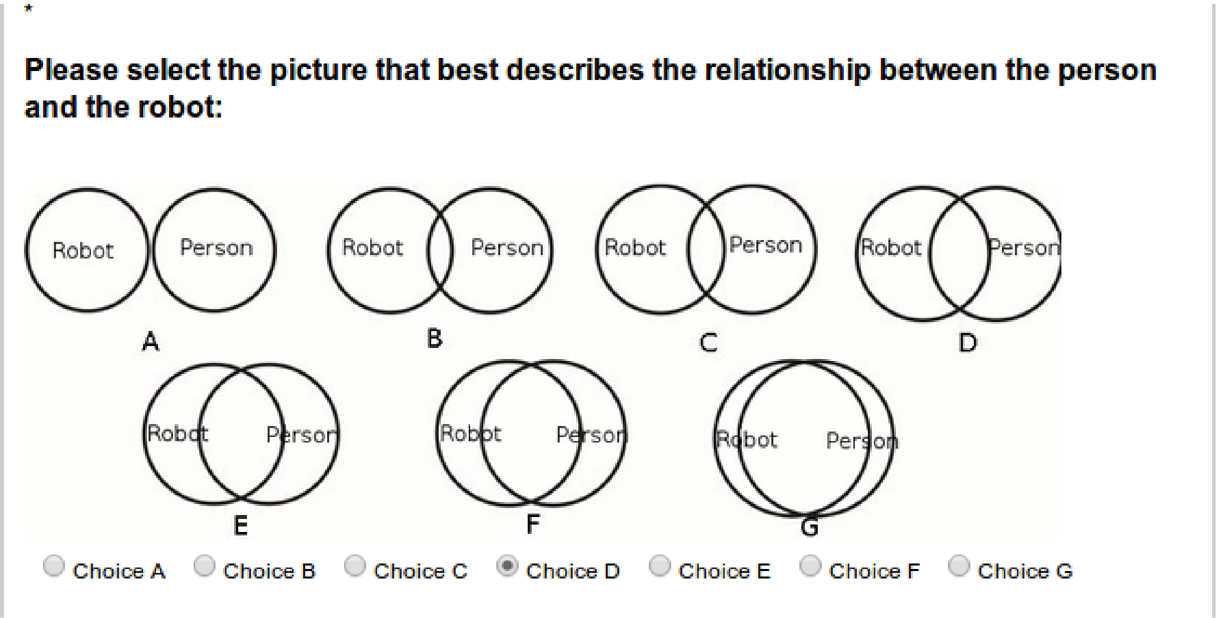

Figure 1: IOS Scale

Analysis

Characteristics of the Sample

There were a 120 participants in the sample. The mean age was 35.28 with a median age of 32. There were 36 females in the sample, and 83 males.

Godspeed Analysis

Reliability In order to ensure that the sets of pairs described in Table 1 could be used as scales, the internal consistency of the two subscales was assessed using Cronbach's alpha.

```
Reliabilitymatrix <- matrix(nrow = 1, ncol = 3)
colnames(Reliabilitymatrix) <- c("Condition", "Dimension", "alpha")
Reliabilitymatrix[1,]<- c("","","")

Session1Matrix <- matrix(nrow=1, ncol=3)
```

[3] ibid. p.597

```r
Session1Matrix[1,]<- c("1","","")
Reliabilitymatrix<- rbind(Reliabilitymatrix, Session1Matrix)


Con1Likeab <-c("X1Dislike.Like", "X1Unfriendly.Friendly", "X1Unkind.Kind",
               "X1Unpleasant.Pleasant","X1Awful.Nice")

Reliabilitymatrix <- rbind(Reliabilitymatrix, Reliability(Con1Likeab, cleandata,
                                                           "Likeability"))


Con1Int <-c("X1Incompetent.Competent", "X1Ignorant.Knowledgeble",
            "X1Irresponsible.Responsible", "X1Unintelligent.Intelligent",
            "X1Foolish.Sensible")
Reliabilitymatrix <- rbind(Reliabilitymatrix, Reliability(Con1Int, cleandata,
                                                           "Perceived Intelligence"))


Session2Matrix <- matrix(nrow=1, ncol=3)
Session2Matrix[1,]<- c("2","","")
Reliabilitymatrix<- rbind(Reliabilitymatrix, Session2Matrix)

Con2Likeab <-c("X2Dislike.Like", "X2Unfriendly.Friendly", "X2Unkind.Kind",
               "X2Unpleasant.Pleasant","X2Awful.Nice")

Reliabilitymatrix <- rbind(Reliabilitymatrix, Reliability(Con2Likeab, cleandata,
                                                           "Likeability"))

Con2Int <-c("X2Incompetent.Competent", "X2Ignorant.Knowledgeble",
            "X2Irresponsible.Responsible", "X2Unintelligent.Intelligent",
            "X2Foolish.Sensible")

Reliabilitymatrix <- rbind(Reliabilitymatrix, Reliability(Con2Int, cleandata,
                                                           "Perceived Intelligence"))

Session3Matrix <- matrix(nrow=1, ncol=3)
Session3Matrix[1,]<- c("3","","")
Reliabilitymatrix<- rbind(Reliabilitymatrix, Session3Matrix)

Con3Likeab <-c("X3Dislike.Like", "X3Unfriendly.Friendly", "X3Unkind.Kind",
               "X3Unpleasant.Pleasant","X3Awful.Nice")
Reliabilitymatrix <- rbind(Reliabilitymatrix, Reliability(Con3Likeab, cleandata,
                                                           "Likeability"))

Con3Int <-c("X3Incompetent.Competent", "X3Ignorant.Knowledgeble",
            "X3Irresponsible.Responsible", "X3Unintelligent.Intelligent",
            "X3Foolish.Sensible")
Reliabilitymatrix <- rbind(Reliabilitymatrix, Reliability(Con3Int, cleandata,
                                                           "Perceived Intelligence"))
Reliabilitymatrix <- Reliabilitymatrix[-1,]
```

```r
kable(Reliabilitymatrix)
```

Condition	Dimension	alpha
1		
	Likeability	0.87
	Perceived Intelligence	0.86
2		
	Likeability	0.93
	Perceived Intelligence	0.88
3		
	Likeability	0.91
	Perceived Intelligence	0.87

Table 2: Godspeed Reliability

The high Cronbach's α across the three conditions shown in Table 2 suggested that we could proceed treating these two dimensions as interval scales as suggested by Bartneck et al.

While each individual *item* in a *semantic differential scale* may be considered ordinal, although these are somewhat different from *Likert scales* in that responses to a semantic differential scales can be considered to be a function of the visual distance that each response option has to each word in the pair. However, treating summative data created based on Classical Test Theory [4], while having some inherent problems, is hardly controversial . For an overview of this approach we refer to [5] or for a more critical view [6]

```
attach(cleandata)
cleandata$Con1Liking <- (X1Dislike.Like + X1Unfriendly.Friendly + X1Unkind.Kind +
                           X1Unpleasant.Pleasant + X1Awful.Nice)/5
cleandata$Con1Intell <- (X1Incompetent.Competent+ X1Ignorant.Knowledgeble +
                           X1Irresponsible.Responsible +
                           X1Unintelligent.Intelligent + X1Foolish.Sensible)/5

cleandata$Con2Liking <- (X2Dislike.Like + X2Unfriendly.Friendly + X2Unkind.Kind +
                           X2Unpleasant.Pleasant + X2Awful.Nice)/5
cleandata$Con2Intell <- (X2Incompetent.Competent+ X2Ignorant.Knowledgeble +
                           X2Irresponsible.Responsible + X2Unintelligent.Intelligent
                         + X2Foolish.Sensible)/5

cleandata$Con3Liking <- (X3Dislike.Like + X3Unfriendly.Friendly + X3Unkind.Kind +
                           X3Unpleasant.Pleasant + X3Awful.Nice)/5
cleandata$Con3Intell <- (X3Incompetent.Competent+ X3Ignorant.Knowledgeble +                           X3
                         + X3Foolish.Sensible)/5
```

[4] Classical Test Theory assumes the following: 1) a given construct(here likeability and perceived intelligence) is measurable through a set of related components ; 2) Particpants are capable of differntiating between grades of intensity of the presence of a given component and and this differentiation can be given a numeric value (score) is assigned; and 3) the sum of scores (total score) represents the construct's value plus random error.

[5] Nunnally, Jum C., Ira H. Bernstein, and Jos MF ten Berge. Psychometric theory. Vol. 226. New York: McGraw-Hill, 1967.

[6] Martinez-Martin, P. (2010). Composite rating scales. Journal of the Neurological Sciences, 289(1), 7-11.

Scale Creation The subscale scores for each dimension for each condition was calculated and all subsequent analyses of the Godspeed subscales were done on the subscales rather than the individual items. While visual assessment of qqplots and the Shapiro-Wilks test do to some extent suggest that Reviewer 1's concerns regarding the distribution of scores were not unfounded, the robustness of the Anova and the t-tests at these participant numbers do not

```
Variab <- c("Con1Liking", "Con2Liking", "Con3Liking", "Con1Intell", "Con2Intell", "Con3Intell")

NormaltestMatrix <- matrix(nrow=1, ncol=2)
for (x in 1:length(Variab)){
  Pertdat  <- as.numeric(unlist(cleandata[Variab[x]]))

  tempmat <- matrix(ncol=2, nrow=1)

  tempmat[1,1] <- round(shapiro.test(Pertdat)$statistic,2)
  tempmat[1,2]<- round(shapiro.test(Pertdat)$p.value,2)

  NormaltestMatrix <- rbind(NormaltestMatrix, tempmat)

}
NormaltestMatrix <- NormaltestMatrix[-1,]
rownames(NormaltestMatrix)<-Variab
colnames(NormaltestMatrix)<- c("W", p)
```

```
kable(NormaltestMatrix)
```

	W	0.08
Con1Liking	0.94	0.00
Con2Liking	0.97	0.01
Con3Liking	0.92	0.00
Con1Intell	0.96	0.00
Con2Intell	0.98	0.03
Con3Intell	0.92	0.00

Table 3: Normality tests for the Godspeed questionnaire

```
for (x in 1:length(Variab)){
  pertdat <- as.numeric(unlist(cleandata[Variab[x]]))

  qqnorm(pertdat);qqline(pertdat)
  }
```

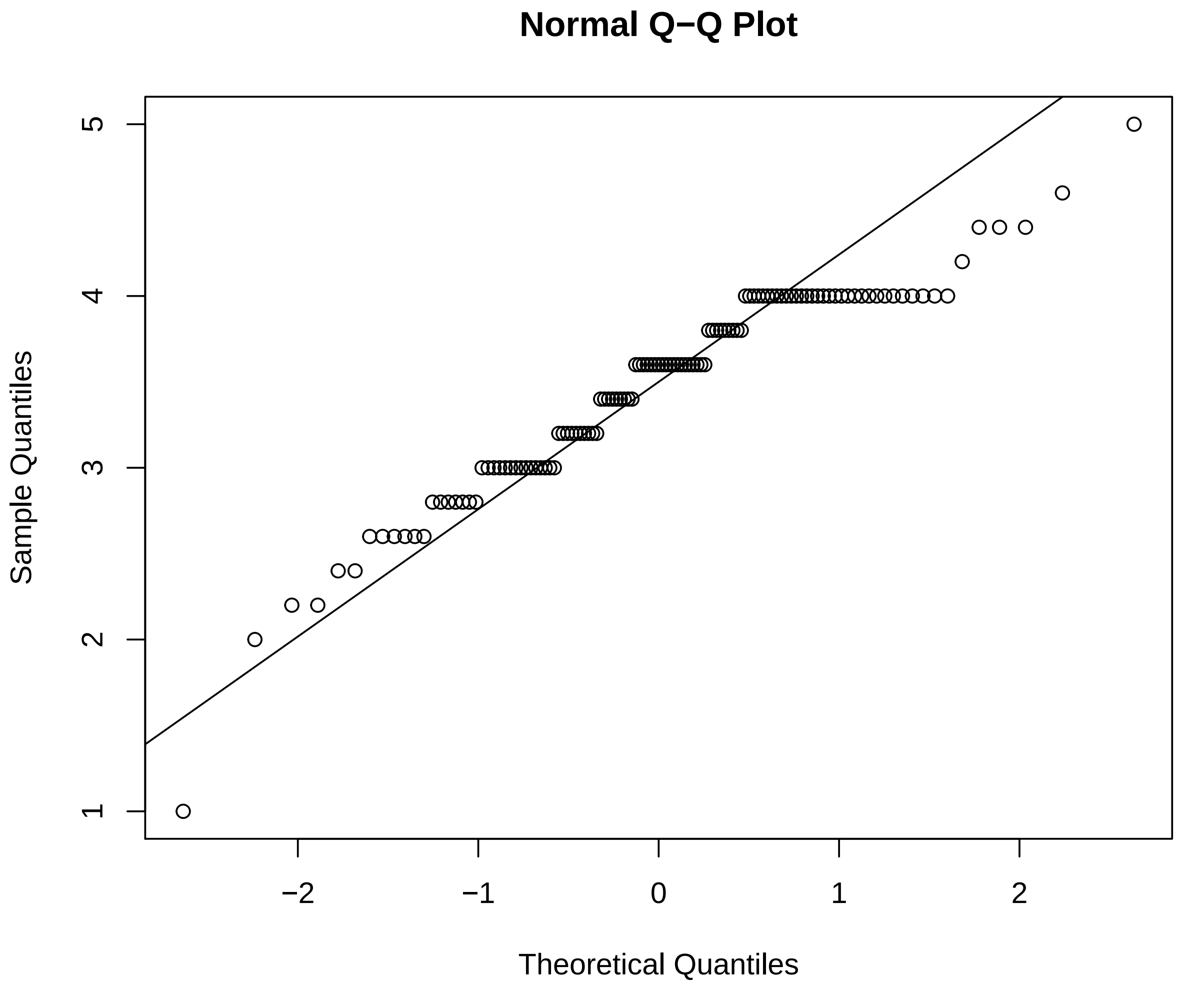
Normal Q−Q Plot
Sample Quantiles
1
2
3
4
5
Theoretical Quantiles
−2
−1
0
1
2

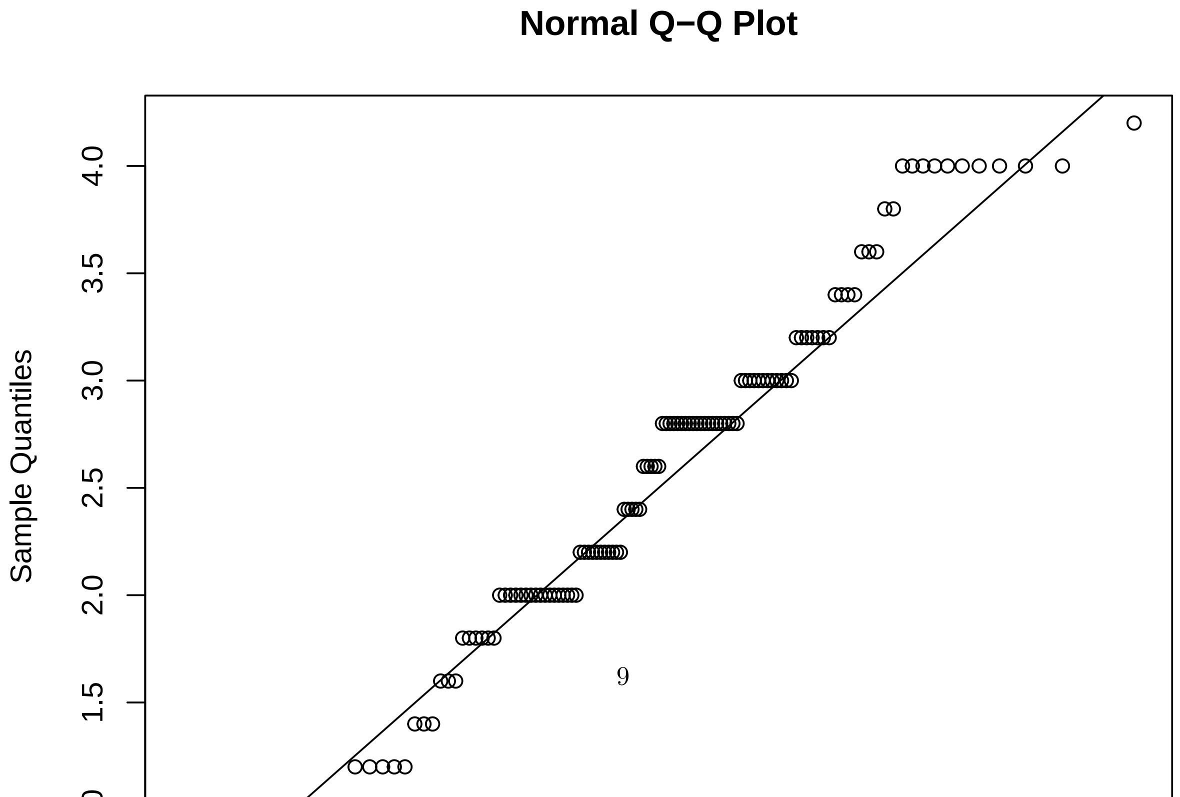
Normal Q−Q Plot
Sample Quantiles
1.5
2.0
2.5
3.0
3.5
4.0

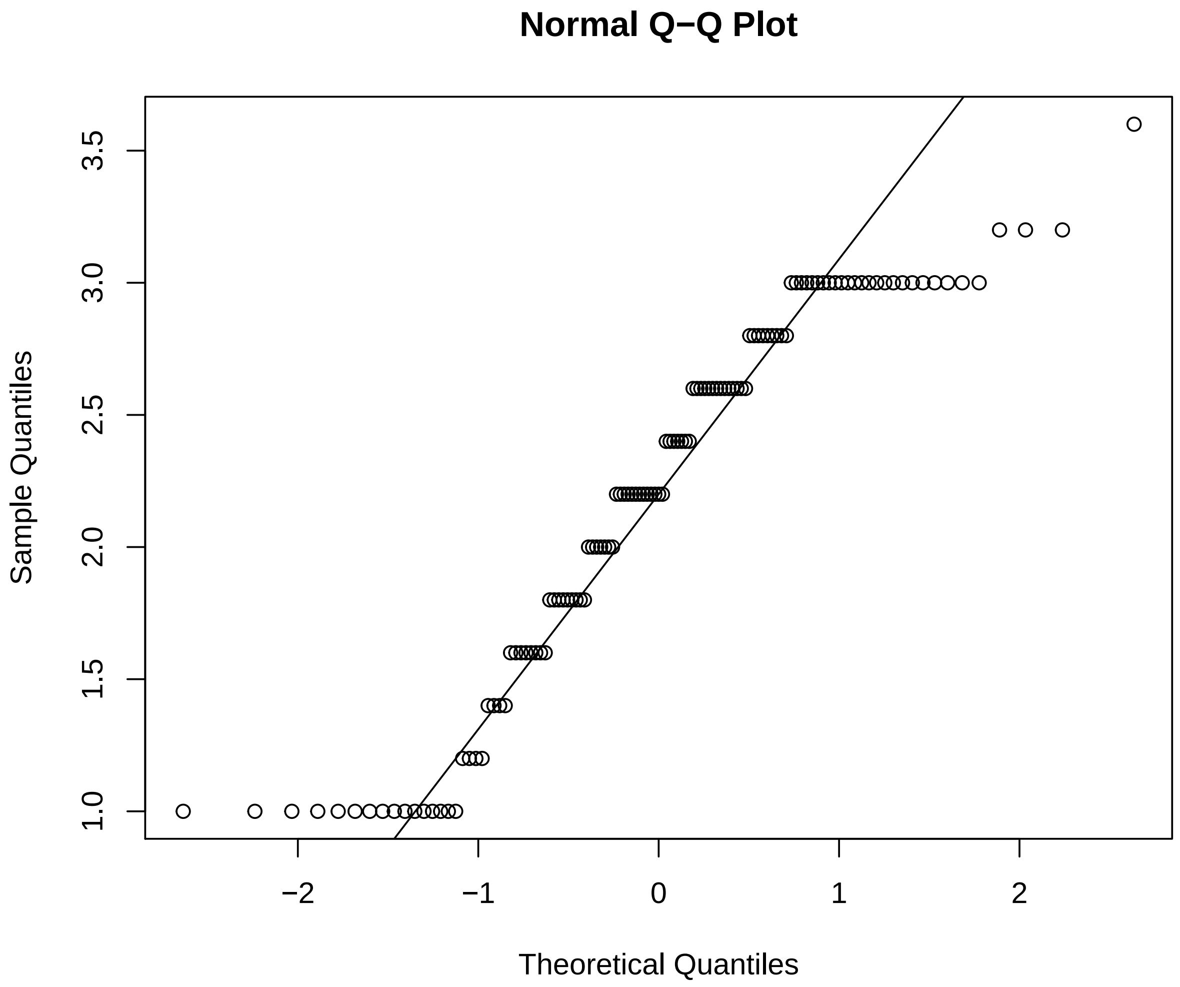

Normal Q−Q Plot
Sample Quantiles
3.5
3.0
2.5
2.0
1.5
1.0
−2
−1
0
1
2
Theoretical Quantiles

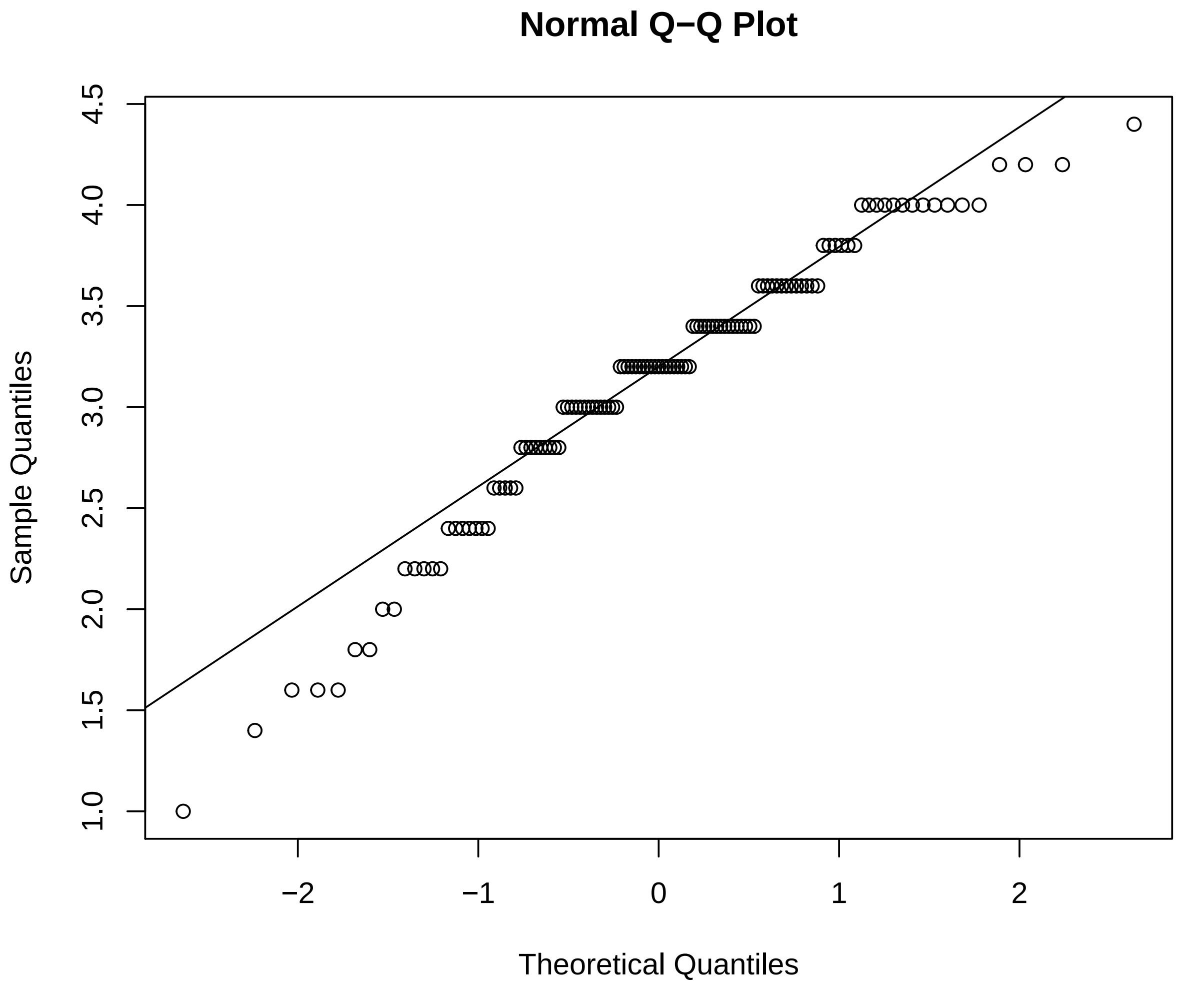

Normal Q−Q Plot
Sample Quantiles
Theoretical Quantiles
1.0
1.5
2.0
2.5
3.0
3.5
4.0
4.5
−2
−1
0
1
2

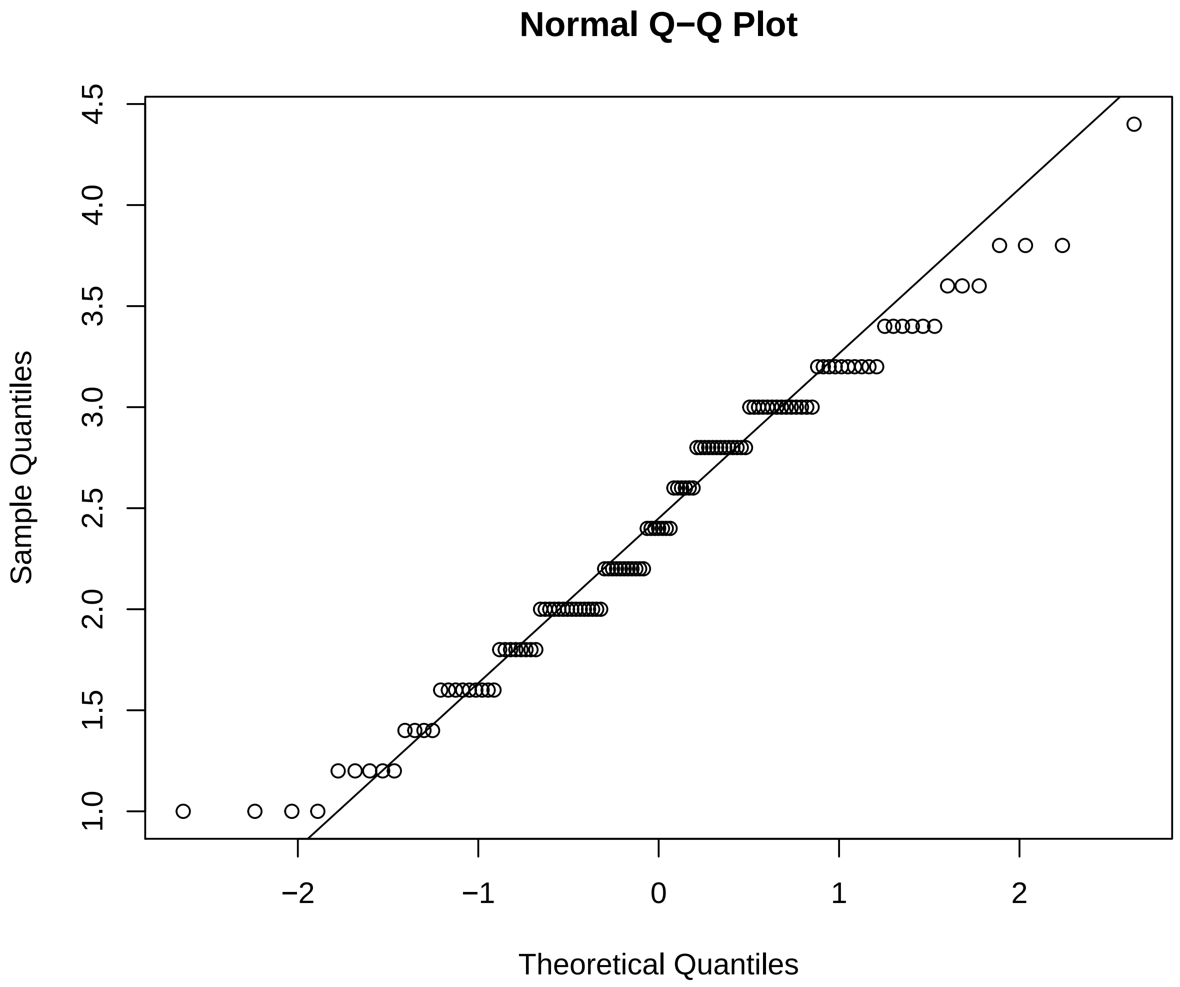
Normal Q−Q Plot
Sample Quantiles
4.5
4.0
3.5
3.0
2.5
2.0
1.5
1.0
−2
−1
0
1
2
Theoretical Quantiles

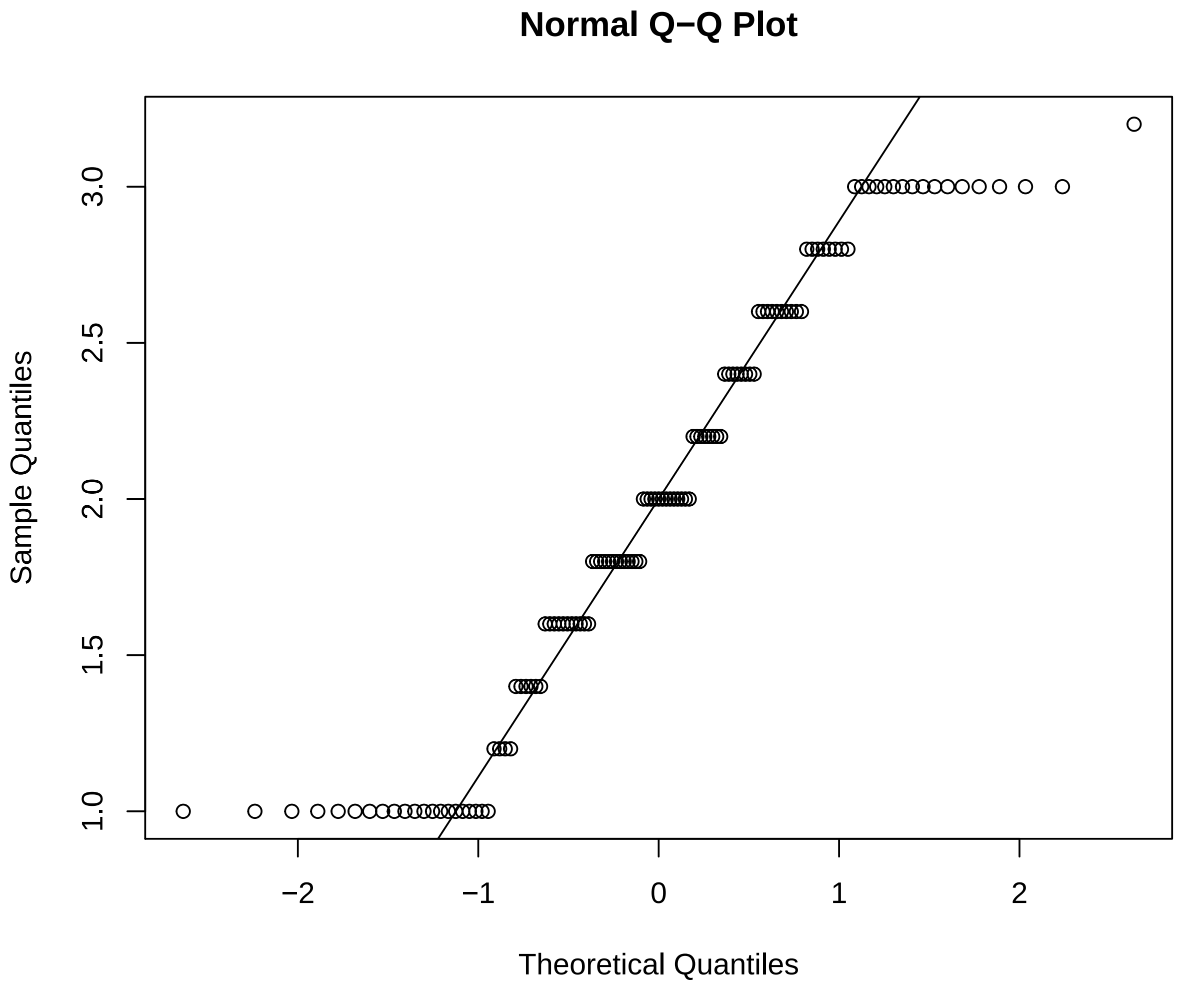

Likeability The descriptive statistics for Likeability are presented in Table 3.

```
LikeabilityVar <- c("Con1Liking", "Con2Liking", "Con3Liking")
Likeabilitynames <- rep("Likeability", times=3)

LikabDesc <- SequenceMatrix(LikeabilityVar, cleandata, Likeabilitynames)
```

```
kable(LikabDesc)
```

Condition	Variable	Mean (SD)	Median	95%CI	t(p)
Condition 1	Likeability	3.47 (0.6)	3.6	3.36 – 3.58	8.48 (0)
Condition 2	Likeability	2.56 (0.81)	2.6	2.41 – 2.71	-5.93 (0)
Condition 3	Likeability	2.2 (0.71)	2.2	2.07 – 2.33	-12.24 (0)

Table 4: Descriptive Statistics Likeability

The results suggest that for the Likeability dimension, participants overall scored the robot higher than a "neutral" score of 3 only in condition 1, while the participants scored the robot lower than this "neutral" score in both condition 2 and 3[^notabene]. The effect of condition on participant ratings along this dimension was assessed using a repeated measures ANOVA.

```
Xmodel<- lm(cbind(cleandata$Con1Liking, cleandata$Con2Liking, cleandata$Con3Liking)~1)
xmatrix <- matrix(nrow = 3, ncol = 1)
colnames(xmatrix) <- ("Condition")
xmatrix[,1]<- c(LikeabilityVar)
xmatrix<-as.data.frame(xmatrix)



AnovaDat<-summary(Anova(Xmodel, idata=xmatrix, idesign=~Condition, type="III"))

AnovaMatrix<-matrix(nrow=1, ncol=4)
colnames(AnovaMatrix) <-c ("df","F", "p", "eta")
ACols <- AnovaDat$univariate.tests
AnovaMatrix[1,1]<- paste(ACols[4], ",", ACols[8])
AnovaMatrix[1,2]<-round(ACols[10],2)
AnovaMatrix[1,3]<-round(ACols[12],2)
AnovaMatrix[1,4]<-round(ACols[2]/(ACols[2]+ACols[6]),2)

Spheric <- AnovaDat$sphericity.tests
```

The repeated measures ANOVA found a significant effect for Conditon(F(2 , 236)=147.12, p=0, partial η^2=0.55. The data did not significantly violate the sphericity assumption (Mauchley 0.97, p= 0.21)

Table Post hoc tests.

```
kable(Posthoc(LikeabilityVar, cleandata))
```

Pair	Mean Difference	95% CI of Diff.	t(df)	p
Con1Liking - Con2Liking	0.91	0.75 – 1.07	11.38(118)	0
Con1Liking - Con3Liking	1.27	1.13 – 1.41	18.17(118)	0
Con2Liking - Con3Liking	0.36	0.21 – 0.52	4.59(118)	0

Table 5: Post hoc tests for Likeability

Posthoc tests presented in Table 4, suggest that there were significant differences between all three conditions, with Condition 1 receiving the highest scores, followed by Condition 2 and Condition 3 receiving the lowest scores.

```
IntelligenceVar <- c("Con1Intell", "Con2Intell", "Con3Intell")
Intelligencnames <- rep("Perceived Intelligence", times=3)

IntellDesc <- SequenceMatrix(IntelligenceVar, cleandata, Intelligencnames)
```

```
kable(IntellDesc)
```

Condition	Variable	Mean (SD)	Median	95%CI	t(p)
Condition 1	Perceived Intelligence	3.13 (0.67)	3.2	3.01 – 3.25	2.15 (0.03)
Condition 2	Perceived Intelligence	2.42 (0.74)	2.4	2.29 – 2.56	-8.51 (0)
Condition 3	Perceived Intelligence	2 (0.69)	2	1.87 – 2.12	-15.92 (0)

Table 6: Descriptive Statistics for Perceived Intelligence

Perceived Intelligence The results presented in Table 5 suggest that, for the Perceived Intelligence dimension as well, participants would only score the robot higher than a "neutral" score of 3 only in condition 1, and lowerthan this "neutral" score in condition 2 and 3. The effect of condition on participant ratings along this dimension was likewise assessed using a repeated measures ANOVA.

```
Xmodel<- lm(cbind(cleandata$Con1Intell, cleandata$Con2Intell, cleandata$Con3Intell)~1)
xmatrix <- matrix(nrow = 3, ncol = 1)
colnames(xmatrix) <- ("Condition")
xmatrix[,1]<- c(IntelligenceVar)
xmatrix<-as.data.frame(xmatrix)



AnovaDat<-summary(Anova(Xmodel, idata=xmatrix, idesign=~Condition, type="III"))

AnovaMatrix<-matrix(nrow=1, ncol=4)
colnames(AnovaMatrix) <-c ("df","F", "p", "eta")
ACols <- AnovaDat$univariate.tests
AnovaMatrix[1,1]<- paste(ACols[4], ",", ACols[8])
AnovaMatrix[1,2]<-round(ACols[10],2)
AnovaMatrix[1,3]<-round(ACols[12],2)
AnovaMatrix[1,4]<-round(ACols[2]/(ACols[2]+ACols[6]),2)

Spheric <- AnovaDat$sphericity.tests
```

The repeated measures ANOVA found a significant effect for Conditon(F(2 , 236)=114.53, p=0, partial η^2=0.49. The data did not significantly violate the sphericity assumption (Mauchley 0.96, p= 0.12)

```
kable(Posthoc(IntelligenceVar, cleandata))
```

Pair	Mean Difference	95% CI of Diff.	t(df)	p
Con1Intell - Con2Intell	0.71	0.55 – 0.86	8.91(118)	0
Con1Intell - Con3Intell	1.13	0.98 – 1.29	14.34(118)	0
Con2Intell - Con3Intell	0.43	0.29 – 0.56	6.27(118)	0

Pair	Mean Difference	95% CI of Diff.	t(df)	p

Table 7: Post hoc tests for Perceived Intelligence

Post hoc tests shown in Table 6 found significant differences between all three conditions, suggesting that participants rated the robot highest along this subscale in condition 1 followed by condition 2 and finally by condition 3.

Inclusion of Other in Self Scale

As discussed above, the nature of the data obtained from the IOS scale may be open for discussion. We will provide the both results from the Friedman's test as well as the repeated measures ANOVA, however, we have settled on using the ANOVA in the paper itself. For the purposes of this analysis, choices were given a rating from 1 to 7, with 1 being the choice with no overlap, and 7 the choice with the most overlap.

```r
attach(cleandata)
```

```
## The following objects are masked from cleandata (position 3):
##
##     Age, Condition1, Condition2, Condition2.1, Experience, Gender,
##     id, IOS1, IOS2, IOS3, Seenit, Status, X1Awful.Nice,
##     X1Dislike.Like, X1Foolish.Sensible, X1Ignorant.Knowledgeble,
##     X1Incompetent.Competent, X1Irresponsible.Responsible,
##     X1Unfriendly.Friendly, X1Unintelligent.Intelligent,
##     X1Unkind.Kind, X1Unpleasant.Pleasant, X2Awful.Nice,
##     X2Dislike.Like, X2Foolish.Sensible, X2Ignorant.Knowledgeble,
##     X2Incompetent.Competent, X2Irresponsible.Responsible,
##     X2Unfriendly.Friendly, X2Unintelligent.Intelligent,
##     X2Unkind.Kind, X2Unpleasant.Pleasant, X3Awful.Nice,
##     X3Dislike.Like, X3Foolish.Sensible, X3Ignorant.Knowledgeble,
##     X3Incompetent.Competent, X3Irresponsible.Responsible,
##     X3Unfriendly.Friendly, X3Unintelligent.Intelligent,
##     X3Unkind.Kind, X3Unpleasant.Pleasant
```

```r
cleandata$IOS1Number[IOS1 == "Choice A"] <- 1
cleandata$IOS1Number[IOS1 == "Choice B"] <- 2
cleandata$IOS1Number[IOS1 == "Choice C"] <- 3
cleandata$IOS1Number[IOS1 == "Choice D"] <- 4
cleandata$IOS1Number[IOS1 == "Choice E"] <- 5
cleandata$IOS1Number[IOS1 == "Choice F"] <- 6
cleandata$IOS1Number[IOS1 == "Choice G"] <- 7



cleandata$IOS2Number[IOS2 == "Choice A"] <- 1
cleandata$IOS2Number[IOS2 == "Choice B"] <- 2
cleandata$IOS2Number[IOS2 == "Choice C"] <- 3
cleandata$IOS2Number[IOS2 == "Choice D"] <- 4
cleandata$IOS2Number[IOS2 == "Choice E"] <- 5
cleandata$IOS2Number[IOS2 == "Choice F"] <- 6
```

```
cleandata$IOS2Number[IOS2 == "Choice G"] <- 7


cleandata$IOS3Number[IOS3 == "Choice A"] <- 1
cleandata$IOS3Number[IOS3 == "Choice B"] <- 2
cleandata$IOS3Number[IOS3 == "Choice C"] <- 3
cleandata$IOS3Number[IOS3 == "Choice D"] <- 4
cleandata$IOS3Number[IOS3 == "Choice E"] <- 5
cleandata$IOS3Number[IOS3 == "Choice F"] <- 6
cleandata$IOS3Number[IOS3 == "Choice G"] <- 7
```

```
IOSVar <- c("IOS1Number", "IOS2Number", "IOS3Number")
IOSnames <- rep("IOS", times=3)

IOSDesc <- IOSSequenceMatrix(IOSVar, cleandata, IOSnames)
```

```
kable(IOSDesc)
```

Condition	Variable	Mean (SD)	Median	95%CI
Condition 1	IOS	2.91 (1.25)	3	2.68 – 3.13
Condition 2	IOS	1.92 (1.06)	2	1.72 – 2.11
Condition 3	IOS	1.17 (0.53)	1	1.07 – 1.26

Table 8: Descriptive Statistics for IOS

IOS ANOVA While the results in Table 7, suggest clear differences between the conditions, all the mean median scores are below the "middle" rating of 4.

```
Xmodel<- lm(cbind(cleandata$IOS1, cleandata$IOS2, cleandata$IOS3)~1)
xmatrix <- matrix(nrow = 3, ncol = 1)
colnames(xmatrix) <- ("Condition")
xmatrix[,1]<- c(IOSVar)
xmatrix<-as.data.frame(xmatrix)



AnovaDat<-summary(Anova(Xmodel, idata=xmatrix, idesign=~Condition, type="III"))

AnovaMatrix<-matrix(nrow=1, ncol=4)
colnames(AnovaMatrix) <-c ("df","F", "p", "eta")
ACols <- AnovaDat$univariate.tests
AnovaMatrix[1,1]<- paste(ACols[4], ",", ACols[8])
AnovaMatrix[1,2]<-round(ACols[10],2)
```

```
AnovaMatrix[1,3]<-round(ACols[12],2)
AnovaMatrix[1,4]<-round(ACols[2]/(ACols[2]+ACols[6]),2)

Spheric <- AnovaDat$sphericity.tests
```

The repeated measures ANOVA found a significant effect for Conditon(F(2 , 236)=130.29, p=0, partial η^2=0.52. The data did, signficiantly deviate from the sphericity assumption (Mauchley 0.88, p= 0). However, the Greenhouse-Geisser correction was quite high, 0.89, so the corrected p value is still well below .01.

```
kable(Posthoc(IOSVar, cleandata))
```

Pair	Mean Difference	95% CI of Diff.	t(df)	p
IOS1Number - IOS2Number	0.99	0.74 – 1.24	7.9(118)	0
IOS1Number - IOS3Number	1.74	1.53 – 1.95	16.45(118)	0
IOS2Number - IOS3Number	0.75	0.55 – 0.94	7.59(118)	0

Table 9: Post hoc tests for IOS

The post hoc tests shown in Table 8 for the IOS scale also suggest the same relationship between conditions as that for the two other measures.

Non-parametric IOS The relationship between the IOS responses and the 3 conditions were also assessed non-parametrically using a Friedman test.

```
IOSData <-cleandata[IOSVar]
IOSData2 <- stack(IOSData)

ID <-rep(1:119,3)
IOSData2[3] = ID
colnames(IOSData2)= c("IOS","Condition","Subject")

IOSFried<-friedman.test(IOS ~ Condition | Subject, data=IOSData2)
IOSDF<-IOSFried$parameter
IOSCH<-round(IOSFried$statistic,2)
IOSp<-round(IOSFried$p.value,2)
```

The Friedman test found signficiant differences between conditions ($\chi^2(2)$=154.06, p=0).

'Post hoc tests' were run using a series of Wilcoxon signed rank tests. These post hoc tests used r as measure of effect size as suggested by Cohen[7] as well as Fritz et al.[8]

```
kable(WilkPosthoc(IOSVar, cleandata))
```

[7] Cohen, J. (1977). Statistical power analysis for the behavioral sciences (rev. Lawrence Erlbaum Associates, Inc.

[8] Fritz, C. O., Morris, P. E., & Richler, J. J. (2012). Effect size estimates: current use, calculations, and interpretation. Journal of Experimental Psychology: General, 141(1), 2.

Pair	Mean Difference	Z	p	r
IOS1Number - IOS2Number	0.99	-6.54	0	-0.6
IOS1Number - IOS3Number	1.74	-9.08	0	-0.83
IOS2Number - IOS3Number	0.75	-6.56	0	-0.6

Table 10: Non-parametric post hoc tests for IOS

The post-hoc tests shown in Table 9 suggest the same relationship as described using the parametric values.
