# Supplementary figures and images for: In Good Company? Perception of Movement Synchrony of a Non-Anthropomorphic Robot
Source: PLoS One. 2015 May 22;10(5):e0127747. doi: 10.1371/journal.pone.0127747 (PMC4441426; doi:10.1371/journal.pone.0127747)

ANNEX 1. Website & Questionnaire


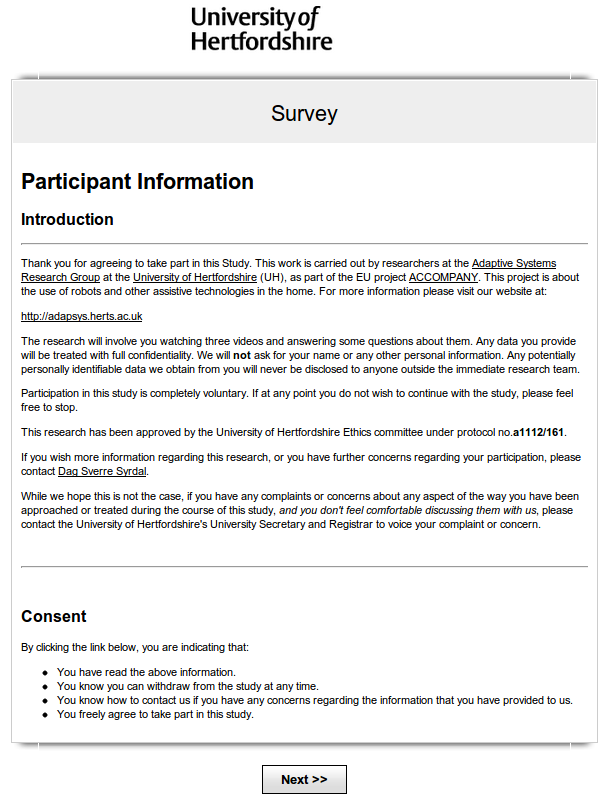


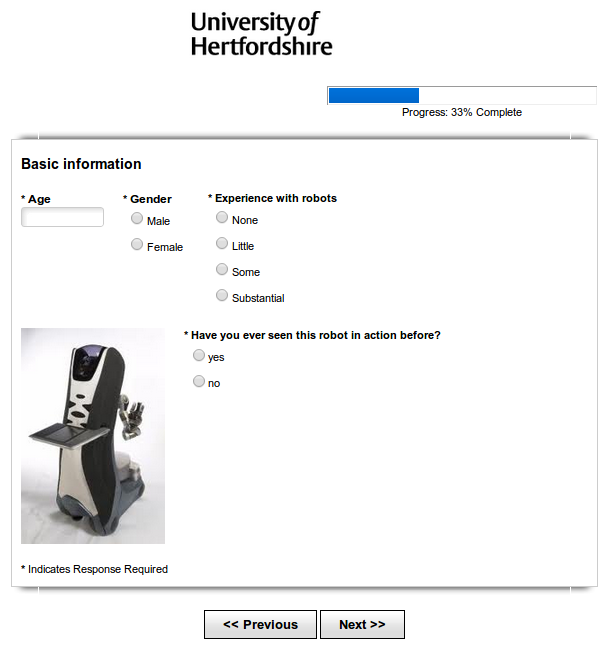


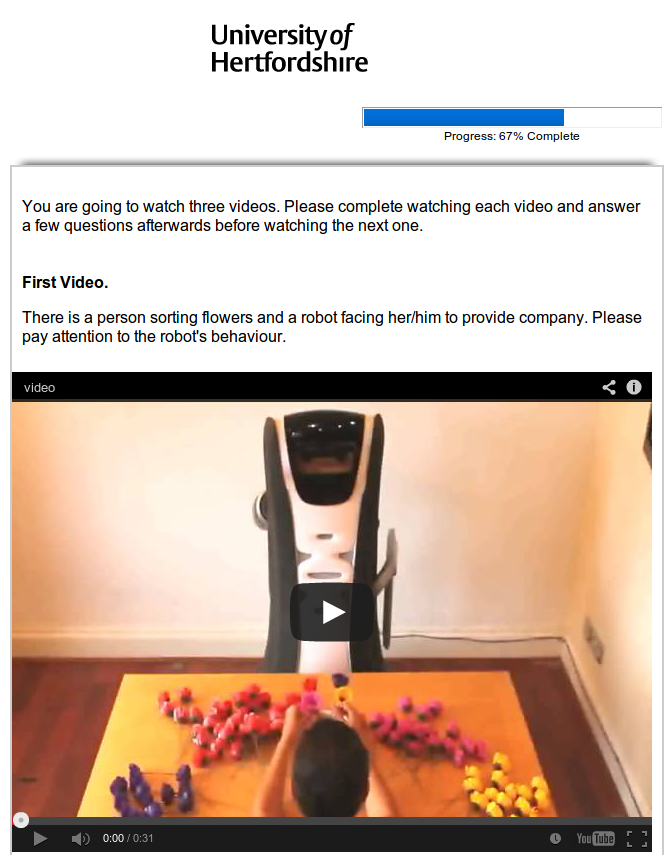


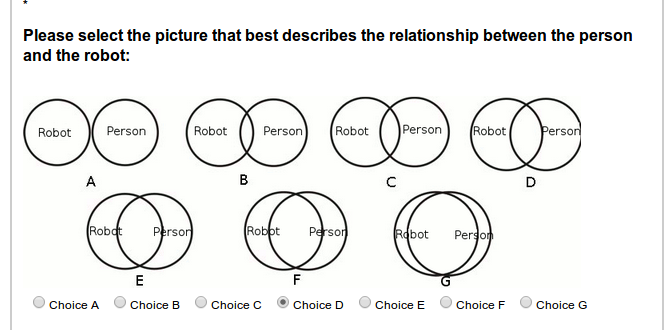


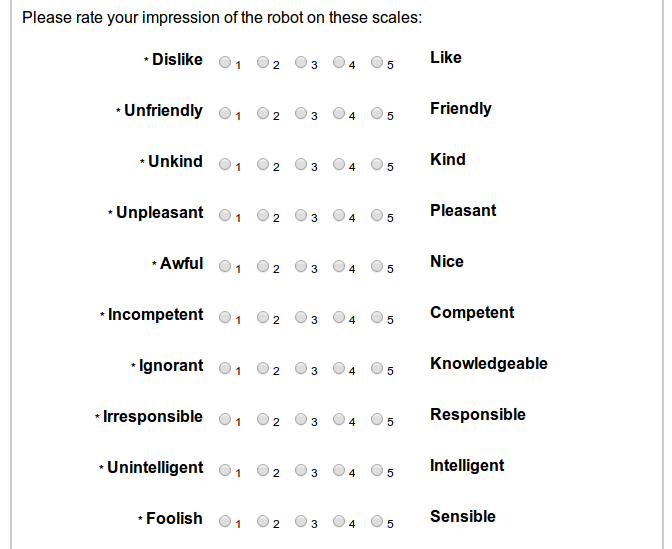

Supplement: S1 Supporting Information — (ZIP) [file pone.0127747.s001.zip › Supporting Information - CompressedZIP File Archive/ANNEX1.docx]
